# Long term endocrine issues in adults born prematurely: a systematic review

**DOI:** 10.3389/fped.2025.1646976

**Published:** 2025-10-30

**Authors:** T. Claffey, A. Cullinan, J. Downey, I. Haupfear, A. Kilbride, E. May, E. Murchan, K. Prendiville, M. Sullivan, J. Trayer, P. Stewart, A. Branagan, E. Roche, J. Meehan, E. J. Molloy

**Affiliations:** ^1^ Discipline of Paediatrics, Trinity College Dublin, the University of Dublin, Dublin, Ireland; ^2^ Paediatrics, Coombe Women’s and Infant’s University Hospital, Dublin, Ireland; ^3^ Trinity Research in Childhood Centre (TRiCC), Dublin, Ireland; ^4^ Endocrinology, Children’s Health Ireland (CHI) at Tallaght, Dublin, Ireland; ^5^ Trinity Translational Medicine Institute (TTMI), St James Hospital, Dublin, Ireland; ^6^ Neonatology, CHI at Crumlin, Dublin, Ireland

**Keywords:** endocrine-, prematurity, long term, thyroid, bone, metabolic, adrenal, reproductive health

## Abstract

**Background:**

Prematurity is a risk factor for chronic disease later in life. According to figures in Ireland, preterm births represent 7% of all births which presents a significant issue for adult healthcare resources. This systematic review synthesised the evidence on long-term endocrine related outcomes for adults who were born prematurely.

**Methods:**

A systematic review was conducted by searching the official databases PubMed and Web of Science. Studies were included in the review based on the criteria that they investigated an endocrine outcome in adulthood in the following categories: issues of the hypothalamic-pituitar*y* axis, growth, thyroid, adrenal function, insulin sensitivity, lipid metabolism, cardiometabolic pathology, and bone health. We were guided by the standards set by the “Preferred Reporting Items for Systematic Review and Meta-Analysis” (PRISMA) Statement.

**Results:**

The search yielded 1,814 studies and after removal of duplicates, 1,584 papers entered screening. 65 full texts were reviewed, after inclusion and exclusion criteria was applied, 27 studies were used for data extraction. Results revealed that being born premature was a significant risk factor for a myriad of endocrine issues in later life. Reduced height, dysfunction of the HPA axis, lower fertility rates, lower bone mineral density and increased odds of hypothyroidism were all outcomes that were associated with preterm birth. Cardiometabolic related outcomes formed the bulk of our data (11/27); these studies found associations between prematurity and increased risk of diabetes, decreased insulin sensitivity, higher body fat percentage and dyslipidaemia.

**Discussion:**

This review highlighted that prematurity is associated with long term endocrine dysfunction in multiple domains. It provided a large set of data demonstrating this association across the various endocrine pathologies relating to bone, thyroid, growth, reproduction and metabolism. This highlights the necessity of long term follow up into adulthood for individuals born preterm.

## Introduction

Globally, approximately 13.4 million babies were born preterm (<37 weeks) in 2020, which accounts for more than 1 in 10 births (1). Furthermore, as the prognosis of extremely preterm infants has improved, the threshold of viability has decreased to 22–24 weeks gestation in many countries. This is a significant shift from 28 weeks gestation in the 1970s (2). In addition to this, there is evidence to suggest that the rate of preterm births is increasing, with rates rising by 12% from 2014 to 2022 in the United States (3). Premature birth is associated with a plethora of long- term conditions in adulthood (4). These chronic disorders involve multiple organ systems, including cardiovascular, endocrine, respiratory, renal, neurodevelopmental and psychiatric (5). With the high prevalence of preterm births, and the fact that this prevalence appears to be increasing, research into the long-term outcomes of this cohort has significant relevance in terms of public health and clinical practice.

This systematic review focused specifically on endocrine conditions affecting adults who were born premature. These endocrine conditions fell into six categories: growth, adrenal, reproductive, bone, metabolic and thyroid health. These categorise were based on the search results and papers were subdivided accordingly. In cases where there were multiple domains they may have been included in multiple areas covered. The aim of this study is to review the prevalence and impact of endocrine issues facing adults who were born prematurely and identify any gaps in the research. We also aimed to outline the effects of these conditions and provide recommendations for their prevention and management.

## Methods

A systematic review of studies found in official electronic databases PubMed and Web of Science was performed to investigate the relationship between prematurity and long term endocrine disorders. PubMed and Web of Science databases were used to search for relevant articles using the search terms: “Outcomes” OR “Consequences” OR “Issues” OR “Dysfunction” OR “Dysregulation” AND “Endocrine” OR “Bone” OR “Hypothalamic pituitar*y* axis” OR “Metabolic” OR “Insulin” OR “Lipid” OR “Cardiometabolic” OR “Growth” OR “Thyroid” OR “Adrenal” AND “Adult born premature” OR “Adult born preterm” NOT “Psychiatric” OR “Neurodevelopmental” OR “Cognitive” OR “Mental health” OR “Neuro-developmental”. All sources were searched on 16/04/2024. This search yielded 1,814 studies (PubMed *n* = 1,435, Web of Science *n* = 379), following the removal of duplicates (*n* = 230) 1,584 papers were identified in total.

The rationale behind the exclusion of neurodevelopmental from our search terms was decided after preliminary searches revealed huge amounts of research linking prematurity to neurodevelopmental outcomes. While we acknowledge that neurodevelopment effects could be related to endocrine issues, we did not include neurodevelopmental as a search term as its not standardly used to describe adult outcomes. In addition the link between and thyroid dysfunction have been recognised for >100 years (6) and therefore did not provide new information on adult outcomes.

Neurodevelopmental was beyond the scope of this review and opted to keep our search terms within the domain of our named endocrine outcomes.

These results were exported to Covidence for shared title and abstract screening. Here, a large volume of studies (*n* = 1,519) were excluded. Criteria required for inclusion were; a history of prematurity (being born before 37 weeks gestation), a population currently in adulthood (selected to be over the age of 17 years and above), and current diagnosis of disease within the scope of the study. If any disputes arose surrounding a paper's inclusion, a separate independent reviewer was used to settle the dispute and finalise its inclusion/exclusion. Narrative and systematic reviews were excluded. Also excluded were papers which did not follow the patient population into adulthood to demonstrate the long-term nature of the disease studied. 65 studies were identified. Studies were then assessed by full text review, where further studies were excluded (*n* = 38). This was due to wrong outcomes (*n* = 27), wrong study design (*n* = 1), and wrong patient population (*n* = 10). Following this, 27 studies were included in our review. Papers included following full text review were then divided by topic: growth, adrenal function, reproductive, bone, metabolic and thyroid.

The outcomes studied varied across each domain of endocrine condition assessed. Texts relating to growth evaluated height and body mass index (BMI), the body weight divided by the square of body height, in relation to premature infants. Studies relating to adrenal endocrine pathology used levels of serum cortisol, Dehydroepiandrosterone (DHEA) sulphate, and androstenedione as primary outcome measures. Texts discussing reproductive health identified infertility as the primary outcome measure, with the probability of reproducing as the main comparative figure between papers. Peripheral quantitative computed tomography was used to measure bone mineral content in the four texts on the topic of bone health in preterm infants. With regards to cardiometabolic, fasting blood glucose, serum insulin levels, and serum lipid levels were used as the measurable outcomes for which data was sought. Studies relating to thyroid pathology identified incidence of hypothyroidism in normal adult populations and populations of those born premature as the primary outcome measure. Abstract screening and full text review was used to identify which domain(s) a paper would fit within.

Tables were assigned to an individual reviewer to analyse the data within the text and transcribe results to a shared online document for data collection. Studies were required to identify preterm populations (infants born before 37 weeks gestation) and fit within the measurable criteria listed above. They must also have had a focus on the effect of these endocrine disturbances within adult life (over seventeen years old). Data was displayed on a table with source, population design, sample size, age at outcome (years), and results as headings consistent to each table.

## Results

**Figure 1:**
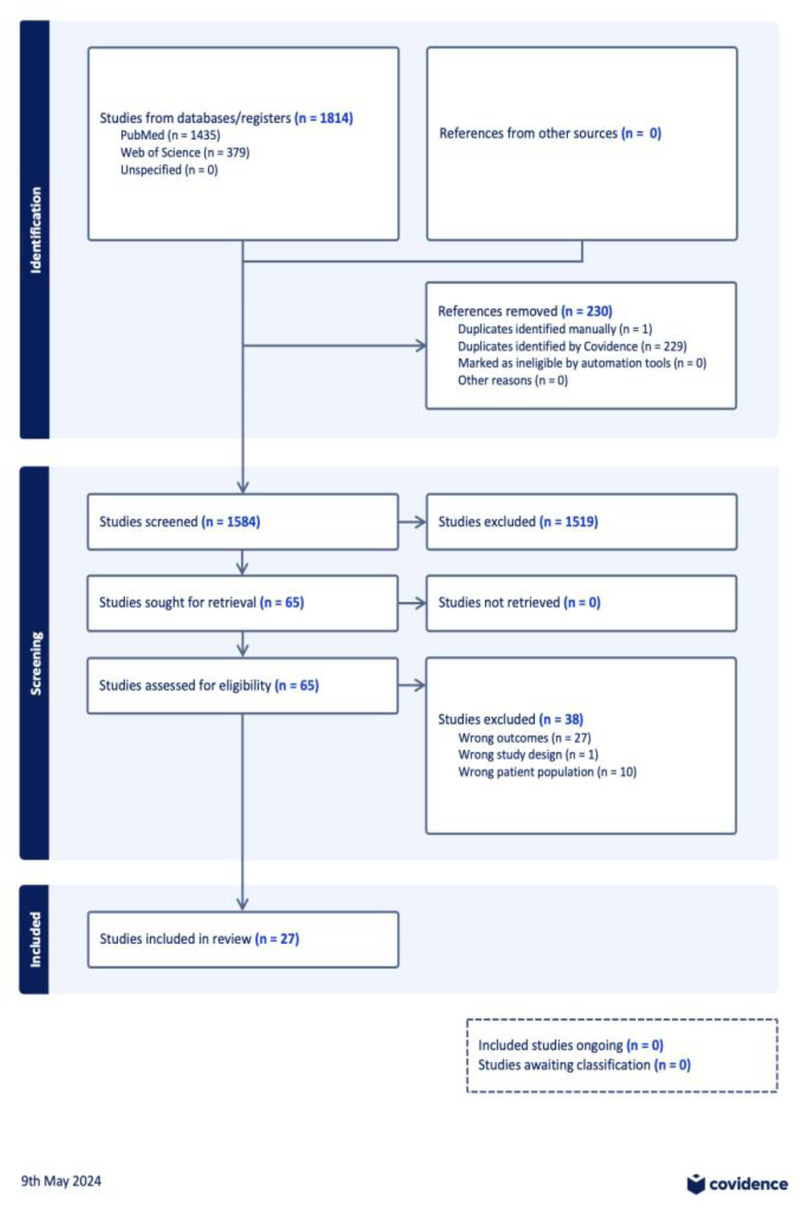
PRISMA flowchart exported from covidence.org.

The search strategy yielded 1,814 studies from the official databases with 1,584 studies entered title and abstract screening, leaving 65 full texts to be analysed for eligibility (Figure 1). After exclusion criteria were applied. Here, 27 studies were included in this systematic review. The majority of the studies that were included were cohort studies (17/27), of these cohort studies 8 were longitudinal. Four (4/27) cross sectional studies were included, and one (1/27) case control study was included. The remainder of the studies were systematic reviews and one randomised control trial. The number of participants ranged, with the largest sample size reaching 1,016,908.

### Growth

Eleven percent (11.1%: 3/27) of studies focused on evaluating the relationship seen between growth and prematurity (7–9). All reviewed articles evaluated height and BMI in relation to premature infants and two thirds of papers (66.6%: 2/3) (7, 8) reported a lower height of premature infants in relation to term infants irrespective of gender, whilst one third (33.3%, 1/3) (9) reported this relationship only in males.

M Hack et al. showed the association between adults born preterm and smaller adult height and BMI in males (8). (Appendix Table A1).

Differences in body composition were assessed by 6 studies (22.2%: 6/27) (10–15). Four studies found that premature infants had higher body fat percentages and/or body mass index (BMI) when compared to their term counterparts (10, 11, 13, 16), while Crane JD et al. reported that the higher BMI's were not statistically significant in their particular study but still found higher levels of adipose tissue surrounding the liver and pancreas in adults born prematurely (14). Alternatively, Rerkasem K et al. found that adults born prematurely were both shorter and weighed less, on average (17). (Appendix Table A5).

### Adrenal function

A study conducted by Meuwese CL et al. (3.7%: 1/27) was found suitable for analysis between prematurity and functioning of the adrenal gland with levels of serum cortisol, DHEA sulphate and androstenedione being the primary outcome measures (18). They reported a significant negative association between levels of DHEAS and prematurity in respect of both genders. Androstenedione concentration levels were shown to be increased in men (but not women) irrespective of administration of corticosteroids (Appendix Table A2).

### Reproductive function

Six of the papers were related to reproductive health (22% 6/27). Of these*,* four papers examined the relationship between being born preterm and infertility in later life. Both females and males born premature displayed a reduced probability of reproducing (19–21). Another by Vikstrom J et al. (22) found that women born with low birth weight (LBW) or small for gestational age (SGA) had an increased risk of infertility. Pandolfi C et al. found that people born with LBW had increased rates of PCOS (23), irrespective of whether they were SGA or appropriate for gestational age (AGA) but premature. James E et al. found no significant difference between the timing of puberty in men and women born preterm or full term (Appendix Table A3).

### Bone results

Four studies which related to bone health of adults who were born premature were found suitable for analysis (11.4%). The four studies used peripheral quantitative computed tomography to measure Bone Mineral Content (BMC) at various sites: hip, lumbar spine, radius, and tibia. Whole body bone area was also measured using dual energy x-ray absorptiometry in one study (24). This study found that VLBW adults had a lower femoral neck Bone Mineral Density, as well as a lower lumbar spine and whole body bone mineral content. Fewtrell et al. found that preterm infants who consumed “almost exclusively” maternal milk had a higher whole body bone area (WBBA) by 3.5% when compared to those who did not (25). Haikerwal et al. found that when compared with controls, young adults born EP/ELBW had lower areal bone mineral density (26). With respect to association between whole body, lumbar and hip bone mineral content (BMC) and prematurity, this was evaluated by one paper by H.A. Weller et al. (33.3%:1/3) (7) which (once corrected for reduced weight and height) was shown not to be associated with prematurity (Appendix Table A4).

### Metabolic results

12 of the 27 studies included in this review were related to metabolic issues including alterations in glucose homeostasis, blood pressure, dyslipidaemia, and body composition in adults born prematurely (44.4%: 12/27). There were 9 papers relating to alterations in glucose homeostasis (29.6%: 8/27) (10–12, 16, 17, 27–30). Two articles found that prematurity had a positive correlation for the development of type 2 diabetes (27, 29), and one article by de Mendonca El et al. found that prematurity had a positive correlation for the development of both type 1 and type 2 diabetes (10).

Regarding other measures of glucose homeostasis, 4 studies (14.8%: 4/27) found associations between prematurity and reduced insulin sensitivity, higher serum insulin levels, or higher fasting blood glucose levels (11, 16, 17, 28). However, Parkinson JRC et al. found no significant difference in fasting glucose and insulin levels between adults born prematurely and those born at term (12), and Pilgaard K et al. also found the same when comparing adults born premature and those who were term-corrected small for gestational age (SGA) at term (29). In relation to prescription of medication for diabetes, one large cohort study *N* = 630,090 conducted by Crump C et al. (30) found that adults born preterm had a higher prevalence of diabetes medication usage or of insulin only medication compared to those who were born at term. In the overall cohort 1.2% were prescribed any medication for diabetes, compared to 1.5%, 1.4% and 1.9% for those born at 35–36, 29–34 and 23–28 weeks respectively. Similar trends were seen for insulin only prescription. Associations between premature birth and cardiometabolic disease were discussed in 3 papers (11%: 3/27) which showed significant incidence of systolic and diastolic hypertension in adulthood when compared to adults born at term (10–12). Four papers (14.8%: 4/27) found that prematurity was associated with higher incidence of dyslipidaemia in adulthood (10–13).

### Thyroid results

Two papers (7.4% 2/27) that were reviewed looked at thyroid outcomes in adults that were born prematurely (31, 32). Both found evidence of increased odds of hypothyroidism in adulthood in those born prematurely when compared to those born at term. Brewer PL et al. looked at multiple conditions such as hypothyroidism, hypertension, and rheumatoid arthritis and found increased odds of developing these conditions on their own or in combination with each other in women born prematurely (31). While this paper addressed incidence rates of rheumatoid arthritis, the relationship between autoimmune diseases and prematurity was outside the scope of this paper (Appendix Table A6).

## Discussion

Our research aimed to analyse the relationship between prematurity and long-term endocrine outcomes in particular in relation to growth, bone health, thyroid and metabolic issues as well as the adrenal and reproductive systems. While the short-term impact of endocrine issues related to prematurity has long formed a major component of neonatal care, the focus on the future impact that prematurity has on endocrine function as an adult has paled in comparison. The results obtained in this systematic review have highlighted the variety of endocrine issues that can manifest in adulthood as a result of prematurity which we believe provides a compelling reason to consider the implementation of expanded scheduled follow-up care of patients who were born preterm extending beyond the current limited routine paediatric follow-up.

Premature babies have in general a lower height as a fully-grown adult in comparison to their term counterparts, although a number of these studies only found this in males. Whole body, lumbar and hip bone mineral content was described as not having an association with prematurity. Prematurity was also shown to have an impact on adrenal function as it was shown, in both males and females, that DHEA sulphate levels were significantly negatively correlated with prematurity, irrespective of weight. Similarly, androstenedione and adione levels were increased only in males regardless of whether corticosteroids were administered.

The reproductive system also has been shown to be impacted by prematurity in relation to fertility. Adult males and females who were born premature experienced a higher risk of infertility which was negatively related to the degree of prematurity i.e., the more premature the person was, the increased chance of infertility. Another individual study illustrated a positive correlation between prematurity and rates of polycystic ovarian syndrome. The timing of puberty was not shown to be impacted by prematurity. The research has highlighted the increased incidence of infertility in adults who were born preterm but does not provide a universal explanation for the underlying mechanism of infertility in these cases. While infertility is commonly multifactorial or indeed found to be without an explanation, one hypothesis could be due to the well-characterised decreased incidence of risk-taking behaviours in adults who were born preterm which could lead to fewer unplanned pregnancies with women born preterm or early term having a lower risk of teenage pregnancy than those born at term (33). Further research is needed to identify if there are underlying biological mechanisms to explain the decreased rates in fertility among ex-preterm adults and while PCOS is a recognised cause of infertility, it is important to identify other potential contributing factors.

Poorer bone health was also found to be associated with prematurity with those born prematurely having a lower femoral neck bone mineral density, lumbar spine and whole body bone mineral content as well as lower areal bone mineral density (height corrected). The bone composition is also shown to be impacted with cortical bone mass in particular being decreased in those born prematurely. It is important to note that the age at outcome for 3 of the studies when followed up for the included studies ranged only from 18 to 25 years. Although in the fourth study (24) the mean age of participants was 29 years, the included studies in general do not address the possibility that prematurity coupled with a natural decline in bone mineral density as seen with ageing, might work synergistically to produce an even more pronounced difference in bone composition in older adults. This is of particular importance because the incidence, morbidity and mortality of falls increases with age - in Ireland the incidence of falls in older adults (aged 70+) is almost 10,500 per 100,000 with a death rate of 54.2 per 100,000 (34). With further longitudinal study of the impact of prematurity on bone health extending into later life, measures to protect against the burdensome impact of osteoporosis and falls of the elderly could be put in place such as routine DEXA screening from a certain age, for example. In addition, given that “almost entirely” breastmilk feeding of preterm infants was associated with increased whole body bone area and bone mineral content in adulthood, this gives strength to the argument that breastmilk is a superior source of nutrition in preterm infants compared to formula feeding with the added benefit in bone health in later life. Advertising the benefits of breastfeeding in campaigns and posters could be a way to increase the uptake of breastfeeding in Ireland.

Research has shown that the thyroid axis is impacted by prematurity. The rates of hypothyroidism, defined non-specifically, in adult life are increased in those who were born prematurely [for twin pregnancies it was found to be associated regardless of the degree of prematurity whereas this was only the case for singleton births that were very preterm (23–31 weeks)]. However, this study (32) focused on those only with medically treated hypothyroidism in adulthood which excludes the potentially significant cohort of adults with subclinical hypothyroidism who were born preterm. Another study (31) found a higher incidence of hypothyroidism in women born prematurely as well as comorbid conditions such as hypertension and/or rheumatoid arthritis. Hypothyroidism and rheumatoid arthritis were the strongest combination. Research is needed as there were only a small number of papers exploring the relationship between prematurity and thyroid conditions. However, given the correlation shown between prematurity and adult hypothyroidism in twins at any gestational age and in singletons born before 31 weeks' gestation, there might be an argument to monitor thyroid function more frequently in twins born prematurely or in those born before 31 weeks' gestation.

Metabolic issues associated with prematurity such as altered glucose homeostasis, type 1 and 2 diabetes mellitus, hypertension, and dyslipidaemia as well as altered body composition were illustrated in a large number of the included studies in this systematic review. Although our aim was primarily focusing on endocrine issues in adults, we found a significant overlap between these features of metabolic syndrome and many of the studies looked at a number of these outcomes. With the complex nature of metabolic diseases, it is difficult to establish prematurity alone to be the primary driver or whether it is just part of a wider range of factors contributing to these diseases such as birth weight for example. An ability to disentangle prematurity from other closely related factors that often are associated with it such as low birth weight would give more strength to the correlation. However, multiple factors may be contributing and we are aware that with all the endocrine issues discussed in this research that prematurity, as well as other factors, may be responsible for the results seen. Nevertheless, our research has strongly suggested prematurity to be a strong driver of the issues described and this important association would benefit from further research and longitudinal studies to establish the true role of prematurity in metabolic disease.

Prematurity and glucose homeostasis was a major topic of discussion in the research reviewed. There was a positive correlation identified between prematurity and both type 1 and 2 diabetes mellitus, as well as reduced insulin sensitivity, elevated fasting blood glucose and serum insulin levels. However, studies with conflicting evidence include research by (12) and (29). For example, an increase in birthweight of 1 kg was associated with a 51% reduced risk of type 2 diabetes (29). This is important to note and our recommendation is for further work to be completed in this area to further establish the relationship.

Rates of hypertension were increased in adults who were premature infants compared to their term counterparts while dyslipidaemia was also found to be more prevalent in this cohort. The majority of the included studies found that body fat percentages and/or BMI were in general higher in ex-premature adults, although one study's results (14) in this area were shown not to be significant while another (17) concluded the opposite - that previously premature adults were shorter and of a lighter weight. Closer weight monitoring and encouraging healthy diet and exercise in patients who were born prematurely could be implemented as not only would this help with weight management but also with optimising their lipid profile and glucose homeostasis. Interestingly (35), research into preterm babies' weight gain in the initial months of life found that preterm infants who gained more weight from birth up to term age had significantly higher body fat percentages, waist circumferences, and acute insulin response in early adulthood compared to those who experienced moderate and low gain in birth weight. Furthermore, a rapid catch-up in weight in the 3 months after reaching term age was associated with higher body fat percentages, waist circumference, and serum triglycerides. Although the focus of our research is on endocrine outcomes in adulthood, it would be remiss of us to fail to consider the correlation illustrated in this study between expedited early neonatal growth in preterm infants and adverse effects in adulthood in relation to elevated body fat percentage, waist circumference and acute insulin response. Methods to monitor premature infants' growth trajectory during the first few months of life to prevent excessive growth could protect against the development of these adverse outcomes in the future but further investigation and longitudinal analysis is required.

There are a number of limitations in the present study that are important to note. These limitations can be broken down into limitations within the review process and limitations within the studies included. There are limitations within the study types selected to be used in this systematic review. Four of the studies selected were cross-sectional, providing data from a single point in time. These studies are useful to show an association between preterm birth and a certain endocrine outcome however, they cannot show causality between the two. To establish this, it would be necessary to have follow up over time in a longitudinal study. Four of the studies selected were retrospective cohort studies. This study design often makes use of historical medical records for information some of which may be inaccurate or incomplete (27). Used the recorded maternal last menstrual period to calculate gestational ages of their cohort born between 1934 and 1944. Additionally, there is potential for recall bias within these studies (36). Eight of the studies selected were longitudinal prospective cohort studies, with the increased demand on resources to complete these studies, are often fewer available to use in systematic review. Inclusion of more studies of this design would strengthen the evidence base of findings between being born preterm and having endocrine problems in later life.

There was a discrepancy in sample sizes among the studies chosen. Six of the studies included in our systematic review contained sample sizes of less than 100 participants, with one including only 35 participants. Due to the limited sample sizes in some of these studies, the statistical power is significantly affected. Many of the studies included upward of 1,000 participants with one study consisting of over one million participants. It is therefore not appropriate to give equal weighting to the findings of studies with such a wide variance in their sample size and thus statistical power. We did not perform any weighting in our analysis and therefore acknowledge this as a limitation that will impact on the conclusions of our review. Two of the three studies selected in the area of growth had an imbalance in the number of males and females included (7). Included 14 males and 36 females with (8) including 1,804 males and 3,894 females. Both of these papers found that height was lower in adults born preterm irrespective of gender, whereas the third paper, with a more equal gender distribution, found that only male height was impacted. This discrepancy highlights a need for further research in the area with a more balanced gender representation to determine the true impact on final height in both males and females. Our review findings are likely affected as a result of this need.

Many of the studies that were used in this review used the terms “very low or low birth weight” interchangeably with or alongside “preterm birth”. An example of such in the area of bone health (26), found that preterm/ low birth weight adults showed signs of impaired bone health at the age of peak bone mass. Other papers looked at those born with a very low birth weight and were also preterm such as (24) who found that this population had a lower femoral neck bone mineral density and lower lumbar spine and whole body bone mineral content. The use of the two terms together in the papers we have selected means it is difficult to ensure that our outcomes of endocrine dysfunction in adulthood are related solely to preterm birth. Mitigation of this limitation would require future research to explore the endocrine effects of preterm birth and low birth weight separately.

Within the review process there was the potential for bias both in terms of selection bias and publication bias. It is important to note the effect of publication bias on the studies that were included in this paper. Van Aert et al. (37) conducted a meta analysis and reported that papers that have significant findings are more likely to be published within medical journals. This means that studies with no significant findings may be unpublished and thus not available to be included in this systematic review. Therefore, there is potential for bias towards a positive association between being born preterm and having endocrine issues in later adult life. It is also important to consider the confounding factor of selection bias. We attempted to reduce this problem by having a clear inclusion criteria, although this could have been further minimised by having a clearly defined exclusion criteria.

There are also limitations with regards to the heterogeneity of the studies included. Notably within the reproductive papers used there was a large variance in the data collection as well as the age of the participants. Of the six papers included, three were retrospective cohort studies, however only one was case controlled. Two of the studies were longitudinal and there was one systematic review. Two studies did not report age at outcome, two studies compared ages to the general population. Two reported participants were between the ages of 20 and 33. Within the 12 papers focusing on metabolic outcomes, three were systematic reviews with meta analyses, three were cross sectional studies, six were cohort studies with three being classified as longitudinal. Four studies only reported age as being over 18 years at time of outcome, six included young adults between the ages of 18 and 30, two studies only included older adults between the ages of 30 and 60. The lack of homogeneity between the reproductive and metabolic studies in particular means making direct comparisons between their results is much more challenging.

We used two scientific databases in our research. These were selected as resources as they include a vast repertoire of research papers and our initial steps of research yielded a significant number of papers that could be screened for potential for inclusion. As a result of this there is potential for relevant studies to have been missed, although significant effort was made to include all papers that were deemed relevant. The use of a greater number of search engines as well as grey literature may have highlighted additional studies and information that may have been relevant to use. Additionally only studies that were written in English were included in the search due to challenges around accurate translation of scientific concepts, this may have led to relevant studies being overlooked. We recognise that limiting our research to two databases could have a possible impact with regards to the translation of the findings of our research to the wider population.

## Conclusion

This systematic review outlined the importance of recognising and addressing the long-term health implications of premature birth, specifically of the endocrine system. To optimise growth and development documentation of both gestational age and birth weight (38), in order to integrate these figures into everyone's core health data. Birth history and the recognition of preterm birth as a risk factor for various lifelong health issues throughout the duration of one's life should not be overlooked, especially as now over 15 million preterm birth survivors annually reach adulthood (6). This highlights the need for continued research on the underlying mechanisms and developing targeted interventions and ensuring follow up of these infants through to their adult lives. Endocrine health complications which these individuals may experience can be more effectively managed by adult clinicians in various health care settings.

## Data Availability

The original contributions presented in the study are included in the article/Supplementary Material, further inquiries can be directed to the corresponding author.

## Appendix A

Search strategy for PubMed was as follows:

(((((((outcomes) OR (consequences)) OR (issues)) OR (dysfunction)) OR (dysregulation)) AND ((((((((((endocrine) OR (bone)) OR (hypothalamic pituitar*y* axis)) OR (metabolic)) OR (insulin)) OR (lipid)) OR (cardiometabolic)) OR (growth)) OR (thyroid)) OR (adrenal))) AND ((adult born premature) OR (adult born preterm))) NOT (((((psychiatric) OR (neurodevelopmental)) OR (cognitive)) OR (mental health)) OR (neuro-developmental)).

Search strategy for Web of Science was as follows:

(((ALL = (adult born premature)) OR ALL = (adult born preterm) AND (((((((((ALL = (endocrine)) OR ALL = (bone)) OR ALL = (hypothalamic pituitar*y* axis)) OR ALL = (metabolic)) OR ALL = (insulin)) OR ALL = (lipid)) OR ALL = (cardiometabolic)) OR ALL = (growth)) OR ALL = (thyroid)) OR ALL = (adrenal) AND ((((ALL = (consequences)) OR ALL = (outcomes)) OR ALL = (issues)) OR ALL = (dysfunction)) OR ALL = (dysregulation)) NOT ((((ALL = (psychiatric)) OR ALL = (neurodevelopmental)) OR ALL = (neuro-developmental)) OR ALL = (cognitive)) OR ALL = (mental health))

**Table A1:** Growth results: data extracted from studies based on growth outcomes.

Source	Sample	Age at outcome (years)	Results
Weiler, 2002 Canada	*N* = 50 subjects = 25 born preterm <1,500 g (7M, 18F all Caucasian) control = 25 born at term matched for age and sex	16–19 y	Shorter height in preterm infants but similar weight and body composition. Negative correlation between time required to regain birth weight and bone mineral content.
Hack, 2003 USA	*n* = 192 (103 M, 92 F) VLBW *N* = 208 (101 M, 107 F) normal birth weight controls.	20 y	No significant difference in weight, height and BMI in VLBW females. VLBW males were significantly smaller at 20 years.
Pfaffle, 2022 Germany	Total *n* = 5,498 infants, preterm = 526 (187 M, 339 F), term birth = 5,172 (1,617 M, 3,555 F)	Females: 16.2 years ± 1.64 and for males:16.8 y ± 1.45	Preterm babies had a lower near final height (NFH). In preterm SGA babies 41% had a NFH <3rd percentile.

**Table A2:** Adrenal results: data extracted from studies based on adrenal outcomes.

Source	Sample	Age at outcome (years)	Results
Meuwese, 2010 Netherlands	*N* = 473 Born 1,983 < 32 weeks preterm; 393 (189M, 204F) Control population of 80 (40 M, 40F) ages 18–25.	19 y	DHEAS shown to be inversely associated with birth weight. Androstenedione levels showed a negative association in women and men, this is with a positive correlation of administration of prenatal betamethasone and postnatal dexamethasone. Serum adione concentration increased in men but not associated with corticosteroid exposure.

**Table A3:** Reproductive results: data extracted from studies based on reproductive outcomes.

Source	Sample	Age at outcome (years)	Results
deKeyser, 2012 Sweden	*N* = 1,016,908: (522,216M, 494,692F) Subjects born preterm = 21,833F, 26,638M	23–33 y	Reduced probability of reproducing in females and males born premature. 5% and >30% lower reproduction in females born <37wk and <27 weeks respectively. In males 6% lower reproductive rates for preterm, 44% lower for extremely preterm.
Van der Pal, 2021 Netherlands	*N* = 370 (male AND female), *N* = 141 female	>18 y	25% of females and 16% of males reported difficulty conceiving. Of 118 pregnancies of firstborn children, 14% used artificial reproduction. Very preterm 25 year old adults had lower reproduction rates than national average.
Van Gendt, 2015 Netherlands	*N* = 293 total (185 female, 108 male)	>18 y	Significantly reduced reproduction rates in females born <32wk (21% vs. control). Males also significantly reduced reproduction rate. Women born preterm more likely to give birth to preterm infant.
Vikstrom, 2014 Sweden	*N* = 1,222	>18 y	Increased risk of infertility in women born LBW or SGA. Risk of being born LBW increased 2.4× in women seeking infertility treatment. Women with female infertility factor were 2.7× more likely to be born SGA.
Pandolfi, 2008 Italy	*N* = 35 LBW (SGA = 19 Premature AGA = 16) Control = 35	21.8 ± 1.4 y	LBW associated with higher risk of developing clinical and biochemical features of PCOS Women who were born SGA/ premature exhibited reduced insulin sensitivity.
James, 2018	N/A	>18 y	Females: 8 studies found no association between preterm birth and timing of menarche. 5 found earlier onset. 1 showed both earlier or later, depending on birth weight. Males: 2 showed earlier puberty. 5 showed no difference. 1 showed later puberty.

**Table A4:** Bone results: data extracted from studies based on bone outcomes.

Source	Sample	Age at outcome (years)	Results
Fewtrell, 2009 UK	*n* = Infants who were born preterm: 43% male, 24% of survivors.	20 y	The extent of human milk in diet positively related with whole body bone area (WBBA) and bone mineral content (BMC). Subjects receiving >90% human milk had higher WBBA and BMC compared to those receiving <10%.
Backstro, 2005 Finland	*N* = 82 {40 adults (17 women and 23 men) born prematurely in the years 1973–1983, who had developed normally	18–27 y	Reduced bone strength index in preterm group, due to smaller cross-sectional size and smaller cortical bone area. Men born preterm had ∼21% lower BSI at tibial shaft than control, no difference in women.
Haikerwal, 2021 Australia	*N* = 291 297 survivors born Extremely Preterm/ELBW and 260 controls (>2,499 g birthweight) (Controls: *n* = 129) (EP/ELBW: *n* = 162).	25 y	Young adults born extremely preterm had lower bone mineral density (BMD) in femoral neck and lower total hip Z-score after adjusting for height and weight. More bone and soft tissue defects seen in males born extremely preterm.
Sandboge, 2022 Finland	*N* = 147 77 Very Low Birth Weight/VLBW participants and 70 controls.	Mean age = 29 y	VLBW adults had 0.25 Z-score unit lower femoral neck BMD, and 0.35 g lower femoral neck bone mineral content (BMC). Lumbar spine and whole-body BMC were lower in VLBW group.

**Table A5:** Metabolic results: data extracted from studies based on metabolic outcomes.

Source	Sample	Age at outcome (years)	Results
Mendonça, 2020	64 studies	>18 y	Positive association between prematurity and developing cardiometabolic disease, with raisedsystolic, diastolic, mean blood pressures, and dyslipidaemia Positive association between prematurity and systemic arterial hypertension shown [OR:1.38 (95% CI: 1.27–1.51)] 1 in 5 studies assessed prematurity and body composition, all reporting increased body fat percentage and BMI in premature infants.
Kajantie, 2010, Finland	*N* = 12,813	>18 y	Preterm infants born before 35 weeks gestation shown to be at increased risk of T2DM [OR:1.68 (95% CI:1.27–1.51)] When adjusting for birthweight this changed to OR:1.59 (95% CI:1.00–2.52)
Kajantie, 2015, Finland	*N* = 207 (107 VLBW cases; 100 control cases)	Mean age 25.0 y	Premature adults born at very low birth weight (VLBW) had moderately lower insulin sensitivity than those born at a similar weight at term.
Markopoulou, 2019	*N* = 43 studies used	>18 y	When comparing adults born premature to adults born at term; Increased body fat contents and cholesterol Increased systolic and diastolic blood pressures Increased fasting glucose and insulin
Pilgaard, 2010, Denmark	*N* = 4,744	Population age range 30–60 y	Premature and SGA babies had similar prevalence of T2DM, hyperglycaemia, hyperinsulinemia, and insulin resistance. Adults born preterm had OR:1.79 (95% CI: 1.17–2.74) for developing T2DM regardless of size at gestational age compared to term babies
Parkinson, 2013, United Kingdom	*N* = 27 studies	>18 y	No significant difference between term and preterm adults in fasting glucose or insulin levels. Preterm birth associated with higher systolic and diastolic blood pressure. Differences more profound in females than males. LDL also raised by a mean 0.14 mmol/L in the preterm group.
Pandolfi, 2008, Italy	*N* = 72 (37 LBW, 35 control)	Mean age 21.4 y	Insulin sensitivity lower in those born at low birth weight in premature and term babies. It was also lower in preterm infants of adequate weight for gestational age than controls.
Morrison, 2016, Canada	*N* = 46 adults born premature with ELBW	Adults age range 22–26 y (participants were followed up at 3, 5, 8, and 12–16 y adulthood)	Adults born premature at extremely low birth weight tended to have higher BMIs, without any statistical significance. They were also noted to have increased adipose tissue surrounding the liver and pancreas, along with increased subcutaneous adipose tissue. These results were not independent to prematurity or birth weight, as sex and BMI were strong predictors.
Rerkasem, 2020, Thailand	*N* = 575 (33 preterm)	Median age 20.6 y	Adults born preterm were 3.2 cm and 6 kg lighter on average Glucose homeostasis was impaired in adults who were born preterm. Insulin resistance and beta cell function were 31% and 43% higher in term adults than preterm.
Breukhoven, 2012, Netherlands	*N* = 455 (167 preterm, 288 term)	Population age range 18–24 y	Preterm adults had increased fat mass. Prematurity was also associated with decreased Lp(a) and increased ApoA-I levels when compared to term adults.
Mathai, 2013, New Zealand	*N* = 52 (31 born preterm)	Mean age 35.7 y	Adults born preterm had more body fat mass than those born at term, with increased abdominal, android, and gynoid fat ratios.
Crump 2011, Sweden	*N* = 630, 090 Preterm = 27, 953	25.5–37 y	Adults born preterm had higher prevalence of diabetes medication use or insulin use compared to those born at term. OR: 1.13 (95% CI 1.02–1.26) for use of diabetes medications.

**Table A6:** Thyroid: data extracted from studies based on thyroid outcomes.

Source	Sample	Age at outcome	Results
Brewer, 2023 United States of America	*N* = 82,514	>18 y	Women born premature (compared to those born at term) ➔ 12% more likely to have hypothyroidism? ➔ 14% more likely to have hypertension ➔ 28% more likely to have rheumatoid arthritis If born prematurely, women are also ➔ 69% more likely to have comorbid hypothyroidism and RA ➔ 48% more likely to have both RA and hypertension ➔ 22% more likely to have hypothyroidism and hypertension. There was a 69% increase in the likelihood of developing all three conditions in women born prematurely.
Crump, 2011 Sweden	*N* = 629,806 (27,935 premature)	>18 y	Adults born prematurely had increased odds of requiring thyroid hormone prescription with odds being even higher in twin births. Twins born premature at greater risk of requiring thyroid hormone replacement (OR: 1.54), and increasing to OR: 2.62 if born between 23 and 31 weeks (relative to those at term). OR: 1.59 (95% Ci 1.18–2.14) singleton births between 23 and 31 weeks requiring thyroid hormone replacement (relative to those at term)
